# Identification of Promising Amaranth (*Amaranthus* spp.) Genotypes Through Multivariate Analysis of Quantitative and Qualitative Descriptors in Boyacá

**DOI:** 10.3390/plants15111648

**Published:** 2026-05-27

**Authors:** Ana Cruz Morillo-Coronado, Ivon Alexandra Díaz-Baquiro, Ana Lucía Pérez-Salamanca

**Affiliations:** 1 Grupo Competitividad, Innovación y Desarrollo Empresarial (CIDE), Facultad de Ciencias Agropecuarias, Universidad Pedagógica y Tecnológica de Colombia, Avenida Central del Norte #39-115, Boyacá 150001, Colombia; ivon.diaz@uptc.edu.co (I.A.D.-B.); ana.perez05@uptc.edu.co (A.L.P.-S.); 2 Grupo Competitividad, Innovación y Desarrollo Empresarial (CIDE), Programa Ingeniería Agronómica, Facultad de Ciencias Agropecuarias, Universidad Pedagógica y Tecnológica de Colombia, Avenida Central del Norte #39-115, Boyacá 150001, Colombia

**Keywords:** *Amaranthus*, morphoagronomic characterization, germplasm, phenotypic diversity

## Abstract

Amaranth (*Amaranthus* spp.) is a pseudocereal with high nutritional value and tolerance to abiotic stresses, essential for food security; however, its use in Boyacá, Colombia, is limited due to a lack of genetic studies. This research aimed to characterize the morpho-agronomic properties of 11 cultivars under greenhouse conditions in Tunja, Boyacá. Six quantitative variables and 19 qualitative descriptors were evaluated using Pearson correlation analysis, principal component analysis, and mixed multivariate clustering. The results revealed a wide phenotypic variability, with leaf width (33.15%) and leaf length (31.21%) showing the highest coefficients of variation. Significant positive correlations were identified between leaf dimensions and plant height, while pigmentation in leaves, stems, and inflorescences proved to be the most discriminating characteristic. The mixed cluster analysis classified the genotypes into six distinct groups, highlighting genotype 10 as the most promising material due to its estimated yield (642.94 kg/ha) and 1000 seed weight (0.91 g), followed by genotypes 2 and 8. It is concluded that there is a high genetic potential in the evaluated materials, and the use of the outstanding genotypes in multi-site field trials is recommended to strengthen regional plant breeding programs.

## 1. Introduction

The genus *Amaranthus* L., belonging to the Amaranthaceae family, comprises a diverse group of approximately 70 species distributed in tropical, subtropical, and temperate regions worldwide [1]. This genus is taxonomically complex due to frequent natural hybridization and extreme phenotypic variability among its members. Three main use categories are distinguished: grain-producing species (*A. hypochondriacus*, *A. cruentus*, and *A. caudatus*), leafy vegetable species (*A. tricolor*), and species classified as competitive weeds (*A. hybridus*, *A. palmeri*) [2].

The resurgence of amaranth as a superfood is not a market trend, but rather a response to its excellent nutritional quality. Unlike true cereals (Poaceae), amaranth grain is a pseudocereal with a protein content ranging from 13% to 18%, surpassing corn and wheat [3]. Most significantly, it boasts a balanced amino acid profile, with high levels of lysine and methionine, amino acids that are often limiting in other grains. Furthermore, its mineral matrix is rich in calcium, magnesium, and highly bioavailable iron. From a nutraceutical perspective, amaranth is a source of squalene (an antioxidant lipid) and bioactive peptides with antihypertensive and anti-inflammatory properties [4,5]. Being naturally gluten-free, it is positioned as a critical ingredient in the food industry for people with celiac disease.

In the current climate change scenario, amaranth emerges as a model of adaptation. By employing the C4 photosynthetic pathway, the plant exhibits a higher CO_2_ assimilation rate under high radiation and temperature conditions than C3 plants (such as wheat or rice). This physiological efficiency translates into remarkable water conservation. Amaranth can close stomata to reduce transpiration without completely halting photosynthesis, which, combined with a taproot system that explores deep soil profiles, grants it exceptional tolerance to prolonged drought [5,6]. Furthermore, osmotic adjustment mechanisms have been documented that allow it to survive in soils with high salinity levels and in high-altitude areas subject to frost, characteristics that make it ideal for the high Andean region of Boyacá in Colombia.

The germplasm of the genus *Amaranthus* has a wide global distribution. According to Schafleitner et al. [7], the largest ex situ collections of amaranth germplasm are at the USDA’s North Central Regional Plant Introduction Station, the National Bureau of Plant Genetic Resources in India, and The World Vegetable Center germplasm bank, with more than 1000 accessions of 13 species. The taxonomic and geographic representativeness in these germplasm banks is remarkable, with the following conservation centers being particularly noteworthy [8]: The United States holds approximately 3300 accessions of *Amaranthus hypochondriacus* from 40 countries. India has 3081 accessions at the National Bureau of Plant Genetic Resources and 2500 accessions of *A. hypochondriacus* at the National Institute of Botanical Research. Peru maintains a genetic nucleus of 740 accessions of *A. caudatus* at the National University of San Antonio Abad in Cusco. However, despite the magnitude of this genetic heritage and the high variability stored, the phenotypic and genotypic characterization of the collections remains incipient. Consequently, amaranth germplasm constitutes an underutilized genetic reservoir with vast unexplored potential for agricultural improvement and food security.

The integration of morpho-agronomic data is a fundamental strategy for the efficient characterization of phenotypic variance in crops. According to Giles et al. [9] this process optimizes the use of germplasm by facilitating the identification and selection of genotypes with superior agronomic attributes, such as productive stability, high yield indices, biotic resistance (to pests and phytopathogens), and resilience to abiotic stresses resulting from climate change and restrictive soil conditions.

Internationally, genetic diversity in *Amaranthus* has been quantified using morphological descriptors, agronomic parameters, physicochemical profiles, and nutritional attributes [6,10]. Recent research highlights the effectiveness of these tools in three main areas. The first is phenotypic discrimination, where Nzungize et al. [11] demonstrated that using 20 qualitative and 12 quantitative descriptors accurately detects intra- and interspecific variability in accessions from Mali. Among the variables with the greatest discriminatory power are leaf pigmentation, inflorescence density, seed coat color, and branching index. The second area is population structure and variability, as exemplified by the study in Ethiopia by Yeshitila et al. [12] who characterized 120 genotypes using 20 qualitative descriptors, concluding that variables such as germination rate, growth habit, presence of axillary spines, sexual expression (dioecious or monoecious) and inflorescence color are determinants for the differentiation of regional germplasm.

Finally, the potential for plant breeding, in studies conducted by Nyasulu et al. [10] in Malawi, confirmed that the use of 13 specific descriptors was statistically significant in defining the uniqueness of the accessions. These results underscore a high selection potential for economically relevant traits, such as leaf and grain biomass yield, as well as earliness, among other fundamental parameters for the selection of elite genotypes.

In Colombia, the situation is paradoxical. Despite having optimal thermal zones and a long-standing Andean agricultural tradition, amaranth has been relegated to a secondary role. National production is marginal and lacks technological advancement, which is reflected in the absence of robust official statistics [13]. Most Colombian scientific literature has focused on amaranth from a weed management perspective (*A. hybridus*), analyzing its herbicide resistance in maize or flower crops [14]. There is a lack of documentation on the morpho-agronomic characterization of cultivars for production purposes in departments like Boyacá. Without local studies in the high Andean zone, producers lack technical recommendations on which genotypes are more productive or resistant to the climatic conditions of the Tunja region.

This research stems from the need to fill this technical gap. Evaluating the phenotypic variability of the materials available in the department is the first critical step toward regional food sovereignty. By identifying high-performing materials under controlled conditions (greenhouse), it is possible to mitigate the risks of biodiversity loss and promote a crop that requires few external inputs.

The overall objective of this work is to characterize the agromorphological germplasm of amaranth (*Amaranthus* spp.) in Tunja, Boyacá. Multivariate analyses (Principal Component and Cluster Analysis) are used to group the 11 evaluated cultivars according to their genetic similarity and yield potential. This study is expected not only to identify superior genotypes, such as the outstanding Genotype 10, but also to serve as a methodological reference for future multi-site field trials, consolidating amaranth as a viable economic alternative for small and medium-sized Colombian producers.

## 2. Results

### 2.1. Morphoagronomic Characterization Using Quantitative Descriptors

The morphoagronomic characterization of amaranth cultivars under greenhouse conditions in Tunja, Boyacá, showed a wide phenotypic dispersion among the 11 genotypes evaluated (Table 1). The analysis of the coefficients of variation (CV) for each material allowed the identification of different levels of intragenotypic homogeneity at a significance level of 95% (*p* < 0.05). Genotype 1 exhibited relative stability in reproductive traits, with a CV of 1.62% for IL and 3.37% for WS. However, foliar parameters showed greater fluctuation, with a CV of 10.79% for LL and 8.73% for PH. Genotype 2 showed moderate variability in leaf architecture (CV of 10.36% for LL, 11.98% for LW), while WS maintained values of 5.23% and 4.46%, respectively. Genotype 3 was the material that presented the least variation in five of the six variables evaluated, with the exception of IL (19.14%).

At the population level, LW emerged as the most plastic trait, exhibiting the highest coefficient of variation (CV = 33.15%), with a mean of 4.23 cm and a range between 1.48 cm (Genotype 7) and 6.67 cm (Genotype 3) (Table 1). This was followed by LL (31.21%) and NL (27.42%). In contrast, traits such as IL (19.14%) and PH (21.21%) showed the lowest CV values, suggesting greater genetic stability or lesser sensitivity to the greenhouse microenvironment.

### 2.2. Correlation Analysis for Quantitative Descriptors

**Figure 1:**
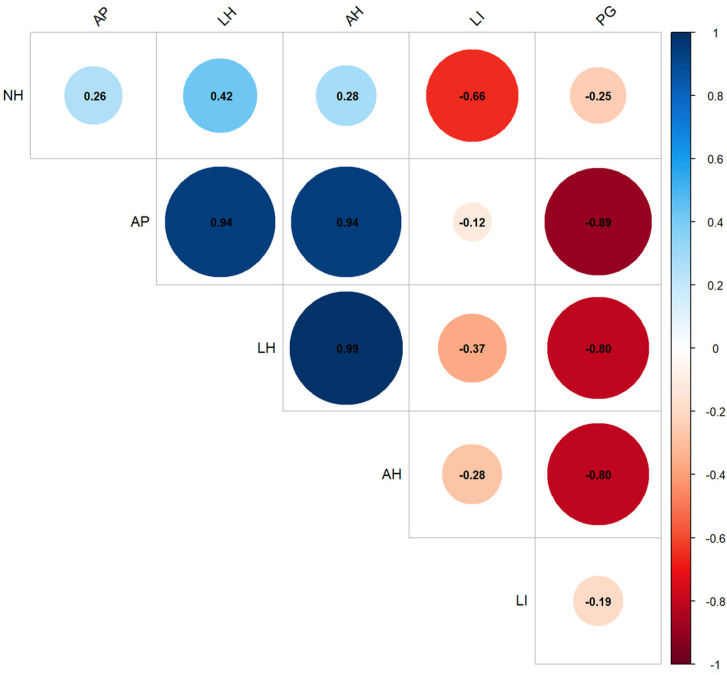
Pearson correlation analysis for the quantitative variables evaluated in the amaranth genotypes. PH: Plant height (cm); NL: Number of leaves; LL: Leaf length (cm); LW: Leaf width (cm); IL: Inflorescence length (cm); WS: Weight of 1000 seeds (g).

As shown in Figure 1, Pearson correlation analysis shows a strong positive correlation between leaf width (LW) and leaf length (LL) with a value of 0.99. Furthermore, there is a positive relationship between PH and LL, as well as between PH and LW, with values of 0.94, respectively. On the other hand, negative but not statistically significant correlations were found between LL and LW (LL and LW) with the WS with values of −0.80, respectively. Similarly, a negative correlation was found between PH and WS, with a value of −0.89. The values obtained in this analysis were significant at the 95% level (*p* < 0.05).

### 2.3. Principal Component Analysis for Quantitative Descriptors

**Figure 2:**
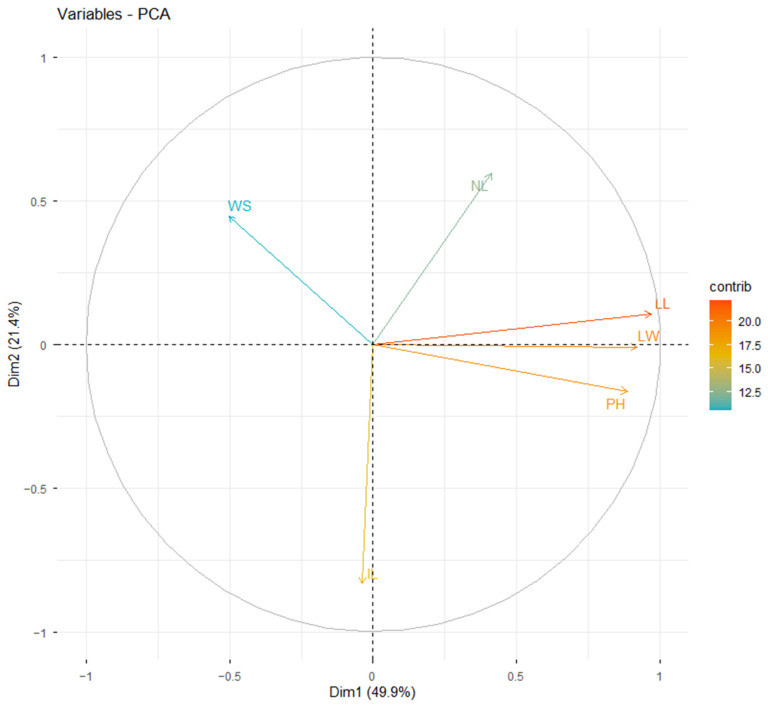
Principal component analysis of the quantitative variables of the amaranth genotypes. PH: Plant height (cm); NL: Number of leaves; LL: Leaf length (cm); LW: Leaf width (cm); IL: Inflorescence length (cm); WS: Weight of 1000 seeds (g).

Principal Component Analysis (PCA) identified the variables with the greatest discriminatory capacity within the evaluated amaranth germplasm. The first principal component (PC1) explained 49.9% of the total phenotypic variance, while the second (PC2) contributed 21.4%. Together, these two orthogonal axes accounted for 71.3% of the cumulative variation (Figure 2), ensuring a reliable two-dimensional representation of genotype dispersion (Table S1).

Vector loading analysis revealed that PC1 captured variance driven by vegetative vigor (PH, LL, and LW), while PC2 reflected leaf development (NL) and reproductive indices (IL), which carried the highest discriminatory weight. The biplot (Figure 2) demonstrated a clear trade-off (inverse relationship) between seed weight (WS) and vegetative vigor, as well as between inflorescence length (IL) and leaf number (NL). In contrast, strong positive associations were confirmed among PH, LL, and LW, with NL also correlates positively with these vegetative traits. These multidimensional patterns confirm a well-structured phenotypic diversity among the 11 amaranth genotypes evaluated in the Tunja (Boyacá) greenhouse trial.

### 2.4. Cluster Analysis for Quantitative Descriptors

**Figure 3:**
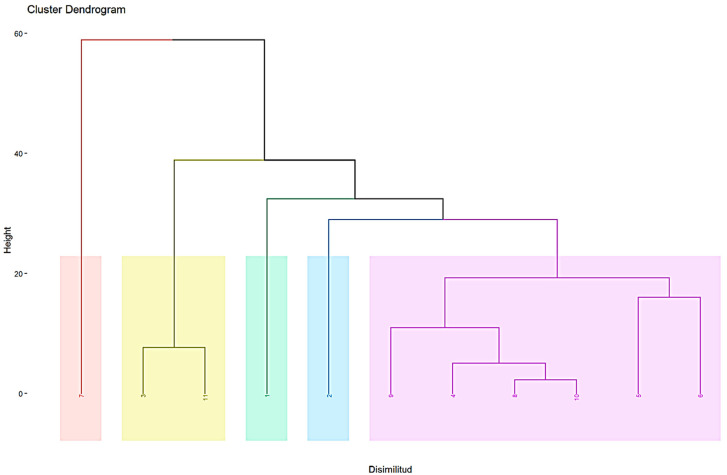
Hierarchical cluster analysis in which the 11 amaranth genotypes were grouped according to quantitative descriptors based on Euclidean distances. Each color represents one of the groups formed: Red: Group I; Yellow: Group II; Green: Group III; Blue: Group IV; and Pink: Group V.

Hierarchical clustering analysis classified the genotypes into five clusters (Figure 3) differentiated by their morphology and yield components:

Cluster I (G7): A divergent group with low height (PH = 36.09 cm) and minimal leaf development (LL = 2.87 cm).

Cluster II (G3, G11): Distinguished by its high vegetative vigor, greater heights (71.33–76.44 cm), and seed weight stability (WS = 0.57 g).

Cluster III (G1): Exhibited low-to-intermediate performance, with superior architecture compared to Cluster I but modest photosynthetic capacity.

Cluster IV (G2): An independent unit characterized by atypical leaf density, registering the highest number of leaves (NL = 73.89).

Cluster V (G4, G5, G6, G8, G9, G10): The most cohesive group, with intermediate and stable morphological characteristics (PH: 55.51–67.39 cm).

The robustness of the five identified clusters was confirmed by the Cophenetic Correlation Coefficient, which reached a value of 0.84, indicating a high fidelity between the dendrogram and the original distance matrix.

In conclusion, clustering allowed for the identification of materials ranging from dwarf varieties to genotypes with high yield potential and unique leaf architectures.

### 2.5. Frequency Analysis of Qualitative Descriptors

The analysis of the qualitative descriptors revealed a wide phenotypic variability among the 11 genotypes evaluated, although with absolute homogeneity in the GH, which was upright (100%) for the entire population (Table 2).

Stems were mostly pubescent (91%) and showed a clear ontogenetic color shift: reddish-green prevailed in the juvenile stage (73%), whereas pinkish-red became most frequent in adult plants (Table 2). Striations were constant, dominated by red (47%) and green (38%) pigments. Leaves were primarily ovate (55%) with entire margins (75%), a rough, prominent surface (82%), and pink petioles (60%). Early-stage leaves typically displayed dark green blades with basal pigmentation in the upper third (45%), which later diversified into green blades with pigmented margins or bases in adulthood.

Reproductive structures were predominantly axillary and apical (81%) with a glomerulate architecture (79%). GH was mostly erect or slightly recurved (91%), and ID was intermediate in 54% of cases. Inflorescence color varied across four phenotypes, chiefly yellow (44%) and purple (22%) (Table 2). Finally, testa seed color (SC) displayed polymorphism, resolving into white (45%), black (36%), and yellow (18%).

**Figure 4:**
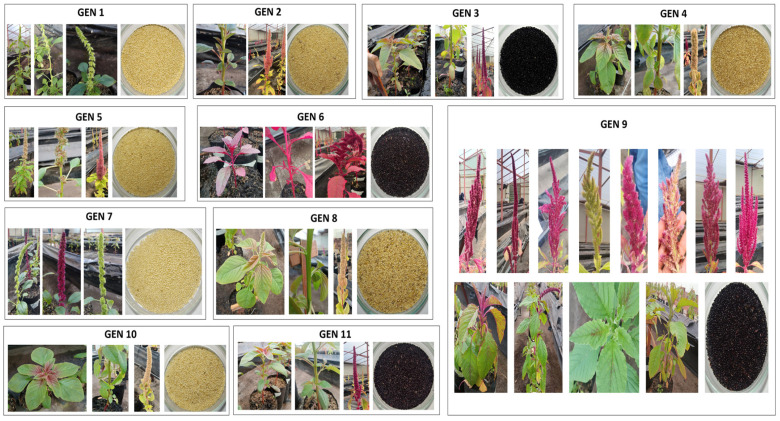
Phenotypic variability observed in the 11 amaranth genotypes evaluated under controlled conditions in Tunja, Boyacá.

Morphological analysis of the 11 evaluated genotypes revealed a predominantly upright architecture, albeit with significant variations in pigmentation and foliar traits that facilitate clear differentiation (Figure 4). Stems ranged from glabrous (genotypes 1 and 7) to pubescent with striations spanning green, red, and purple hues; they also exhibited distinct ontogenetic chromatic shifts from juvenile reddish-green to adult yellow, pink, or purple tones. Regarding leaf morphology, elliptical and ovate shapes predominated, with textures varying from smooth to markedly rough. Petioles were mostly pink, whereas leaf blades transitioned from dark green during youth to pigmented bases or margins at maturity. Inflorescences served as a key diagnostic feature, occurring mainly in axillary and apical positions with glomerulate or amarantiform structures. Their densities fluctuated from medium to dense, displaying a color palette of yellow, pink, purple, and reddish-pink. Finally, seed color acted as a definitive distinctive marker, categorizing the genotypes into white (1, 2, 7, 8, and 10), yellow (4 and 5), and black (3, 6, 9, and 11) groups. This polymorphism reflects a broad genetic and phenotypic diversity within the germplasm, which is essential for future selection and breeding programs.

### 2.6. Multiple Correspondence Analysis for Qualitative Variables

**Figure 5:**
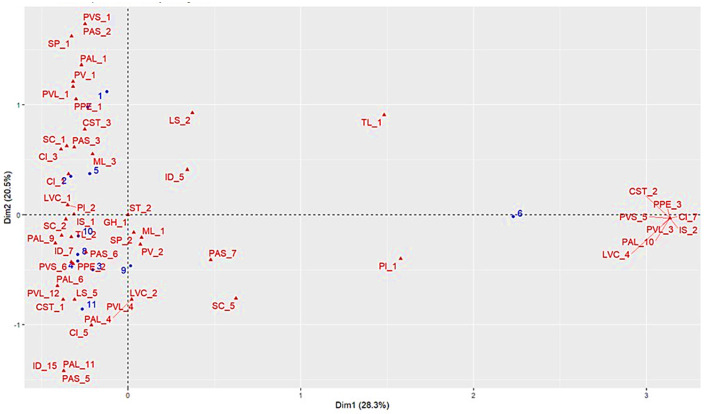
Multiple correspondence analysis of the contribution of qualitative variables. Red letters are the qualitative variables analyzed, numbers in blue are the 11 genotypes studied.

Multiple Correspondence Analysis (MCA) for qualitative variables revealed that the first two dimensions accounted for 49% of the total inertia, with Dim 1 explaining 28.3% and Dim 2 contributing 20.5% (Table S2). Although most of the studied materials shared a common phenotypic background, variables with high diagnostic value were clearly identified at the extremes of both dimensions (Figure 5).

Dimension 1 was primarily defined by pigmentation and leaf/reproductive morphological traits. The categories exhibiting the greatest discriminatory power included vein coloration (LVC_4), petiole coloration (PPE_3), stem coloration (PYS_5), and inflorescence shape (IS_2). Meanwhile, Dimension 2 segregated the materials according to specific color and developmental stages, particularly regarding the pigmentation of both mature (PAS) and young stems (PYS). Finally, while most materials clustered near the centroid—indicating high phenotypic homogeneity—Genotype 6 projected distally as an outlier. Its qualitative profile, characterized by purple pigmentation and a distinctive inflorescence type, markedly separated it from the average phenotype of the evaluated population.

### 2.7. Cluster Analysis for Qualitative Descriptors

**Figure 6:**
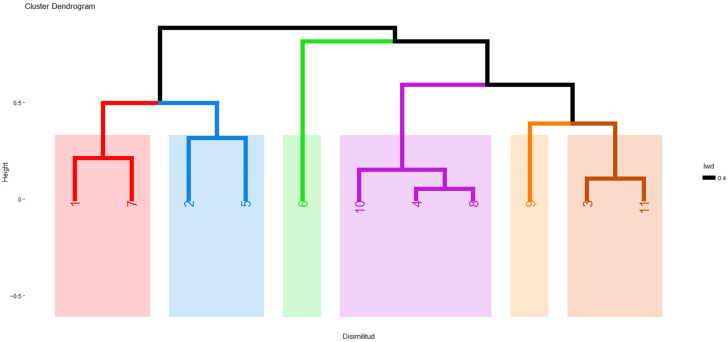
Cluster analysis of the qualitative characters evaluated in the 11 amaranth genotypes using Euclidean distance. Each color represents one of the groups formed as follows: Red: Group I; Blue: Group II; Green: Group III; Purple: Group IV; Group V: Cream; Group VI: Brown.

Hierarchical clustering analysis based on qualitative descriptors classified the 11 genotypes into six main groups (Figure 6) according to their pigmentation patterns and morphology:

Group I (G1, G7): Characterized by a low presence of accessory pigments; they have green foliage, stems that mature from green to yellow, yellow inflorescences, and white seeds.

Group II (G2, G5): They exhibit intermediate pigmentation, with reddish juvenile stems, pink petioles, and pink, erect inflorescences.

Group III (G6): This is the most divergent material (outlier). It stands out for its intense expression of betaines and anthocyanins in all its organs (stem, leaves, and purple/pink flowers) and has black seeds.

Group IV (G4, G8, G10): Genotypes with vegetative vigor and phenotypic stability; They share stems with red streaks, ovate leaves, and dense, glomerulated inflorescences.

Group V (G9): A single cluster defined by its high plasticity and polymorphism. It exhibits a wide range of stem and leaf spot colors and is the only group with both glomerulated and amaranth-like inflorescences.

Group VI (G3, G11): Includes materials with pigments in reproductive structures, characterized by stems with red streaks, dense purple inflorescences, and black seeds.

In Supplementary Table S1, a summary of the main morphological characteristics that defined the groupings of the 11 evaluated amaranth genotypes can be seen.

The robustness of the six identified groups was validated using the Cophenetic Correlation Coefficient (CCC), which yielded a value of 0.86, demonstrating high consistency between the dendrogram and the qualitative similarity matrix. The significance of the separation between clusters was corroborated by a Bootstrap test with 1000 replicates, where the main nodes showed support values greater than 80%, ensuring that the classification is not due to chance, but rather to biological patterns of pigmentation and morphology.

In conclusion, this classification is consistent with Multiple Correspondence Analysis, highlighting Genotypes 6 and 9 as the most unique phenotypic profiles, most distinct from the population average (Table S4).

### 2.8. Mixed Multivariate Analysis

**Figure 7:**
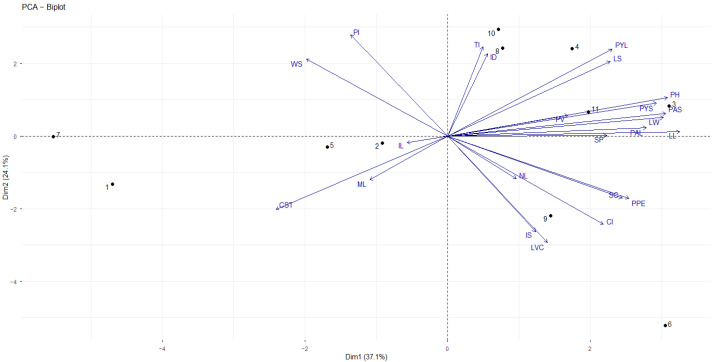
Analysis of mixed factors, taking into account the contribution of the quantitative and qualitative descriptors in the first two components.

Multiple Factor Analysis (MFA) integrated both categorical and continuous variables (Table S3), with the first two dimensions explaining 61.2% of the total phenotypic variance (Figure 7).

Dimension 1 (37.1%) was characterized by qualitative pigmentation descriptors (associated with betalains and anthocyanins), with the horizontal separation of genotypes being mainly driven by leaf, stem, and inflorescence color. Conversely, Dimension 2 (24.1%) was primarily influenced by morphometric variables such as plant height (PH) and leaf dimensions (LL, LW), alongside stem pigmentation patterns. Based on this integration, genotypes G1 and G7 were characterized by short stature and a lack of complex pigmentation (predominantly green and yellow tones). In contrast, G6 exhibited a highly diverse chromatic profile, featuring intense purple pigmentation across all organs. Genotypes G3, G4, G8, G10, and G11 formed the cluster with the greatest vegetative vigor, standing out for their maximum heights and leaf dimensions. Meanwhile, G9 was distinguished by its unique morphometry and the lowest grain yield (0.38 g), whereas G2 and G5 displayed balanced, intermediate phenotypes. Overall, this multi-trait analysis demonstrates that plant height, seed yield, and pigmentation intensity are the fundamental axes defining the genetic divergence of these materials, thereby facilitating strategic selection based on target vigor or coloration traits.

### 2.9. Mixed Cluster Analysis

**Figure 8:**
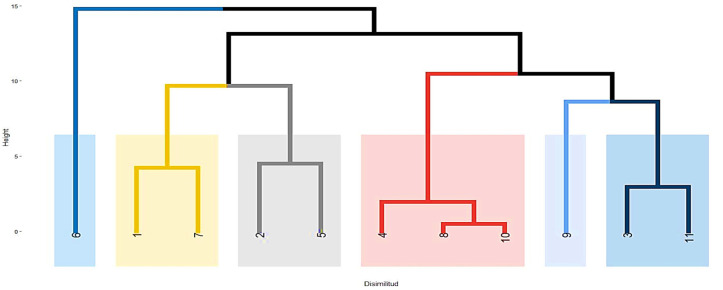
Cluster analysis in which the groups in which the amaranth cultivars are classified are observed according to the qualitative and quantitative variables, using the Euclidean distance. Each color represents one of the six groups. Blue: Group I; Yellow: Group II; Gray: Group III; Red: Group IV; Light Purple: Group V; Dark Blue: Group VI.

The mixed hierarchical clustering analysis integrated quantitative and qualitative variables, classifying the 11 genotypes into six groups differentiated (Figure 8) by their morphology, pigmentation, and yield:

Group I (G6): The most divergent material due to its intense purple pigmentation in stems and leaves, amaranth-colored petioles, and terminal amaranth-like inflorescences.

Group II (G1, G7): Plants with low vegetative vigor and a predominance of green and yellow tones; they produce white seeds and yellow inflorescences.

Group III (G2, G5): They exhibit intermediate morphological development, with elliptical leaves, reddish stems with green streaks, and pink inflorescences.

Group IV (G4, G8, G10): The group with the greatest stability and vigor. They are distinguished by their pinkish-red stems, consistent red streaks, and dense yellow inflorescences.

Group V (G9): Distinguished by its low productivity, registering the lowest thousand-seed weight (0.38 g), and exhibiting pink veins.

Group VI (G3, G11): Includes the tallest plants with the longest inflorescences, characterized by dense purple inflorescences and black seeds.

The robustness of the mixed clustering analysis was validated by the Cophenetic Correlation Coefficient, which yielded a value of 0.82, confirming a faithful representation of the combined distances within the dendrogram structure. Furthermore, bootstrap resampling with 1000 iterations provided node support values exceeding 75% for most major branches, ensuring that the classification of the 11 genotypes is stable and statistically reliable for breeding programs. Consequently, this multivariate integration allowed for a precise separation of genotypes, successfully identifying materials ranging from those with high agronomic potential (Groups IV and VI) to those with unique chromatic profiles (Group I).

Supplementary Table S5 summarizes the main quantitative and qualitative variables that define the groupings of the 11 genotypes evaluated.

### 2.10. Selection of Superior Amaranth Genotypes

For the selection of superior genotypes, the total weight of the seeds obtained in the 9 plants of each genotype was considered, which was extrapolated to a planting density used in commercial crops in Mexico of 260,417 plants/ha [15]. From this estimate, the potential yield of each genotype was determined.

**Figure 9:**
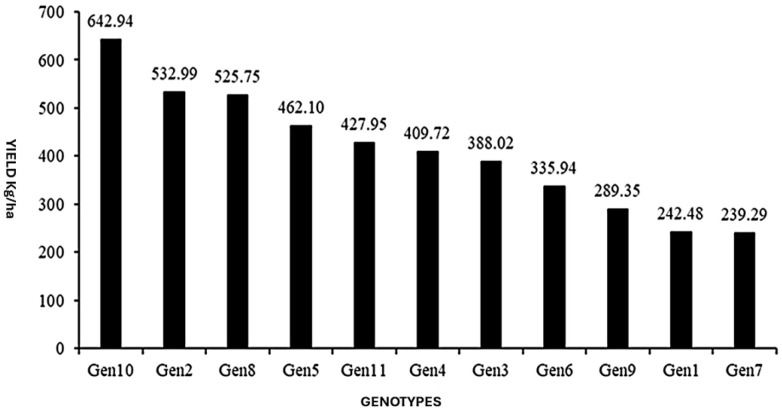
Comparison of seed yield (kg/ha), evaluated in eleven amaranth genotypes.

The performance analysis for the selection of the best genotypes showed significant variations between the evaluated materials. Genotype 10 stood out for obtaining the highest yield (642.94 kg/ha), followed by genotypes 2 and 8 with values of 532.99 kg/ha and 525.75 kg/ha, respectively (Figure 9). Genotypes 5, 11 and 4 showed an intermediate performance (range between 409.72 kg/ha to 462.10 kg/ha), while genotypes 3, 6 and 9 showed a lower performance. Finally, the lowest productive indices were recorded in genotypes 1 and 7 with yields of 242.48 kg/ha and 239.29 kg/ha, respectively.

Additionally, for the identification of promising genotypes, performance was analyzed comprehensively with complementary variables such as PH, IL and WS. In this sense, genotype 10 presented an outstanding agronomic profile by combining the highest productivity with the maximum value of WS = 0.91 g and an PH = 64 cm (Table 3); genotype 2 stood out for its leaf architecture, recording the highest NL in the collection (73.89) and an IL of 29.59 cm; genotype 8 showed consistent values in PH = 62.36 cm and WS = 0.82 g.

In contrast, a direct correlation was observed between low performance and the reduction in biometric variables in genotypes 1 and 7, which presented the minimum PH values (38.19 and 36.09 cm, respectively), as well as reduced dimensions in leaf length and width (LL and LW).

## 3. Discussion

The descriptive analysis of amaranth cultivars, under controlled conditions in Tunja, Boyacá, revealed significant heterogeneity in the six quantitative descriptors evaluated. Morphological characters related to the photosynthetic area, specifically LW (CV: 33.15%), LL (CV: 31.21%) and NL (CV: 27.42%), exhibited the greatest phenotypic plasticity (Table 3). In contrast, IL and PH showed relative stability, with a coefficient variation of 19.14% and 21.21%, respectively (Table 1).

It is important to note that the reference studies found on agromorphological characterization of amaranth have been carried out in open field conditions, at temperatures higher than those of Tunja (average temperature 16 °C), so the greenhouse study helps us to control this environmental variable a little and the results, although compared with field studies, allow us to have an approximate idea of the agronomic behavior that the evaluated genotypes may have under similar conditions.

This variability in leaf characteristics is consistent with what was reported by Sefasi et al. [16], who identified a wide fluctuation in leaf length (CV: 59.5%) and width (CV: 44.5%). However, unlike the present study, these authors also observed variations in inflorescence length (56.2%) and plant height (44.1%) (Table 1). On the other hand, the results of this research present greater similarities with the findings of Kpocheme et al. [17], who reported a pronounced variability in the number of leaves (25%) and leaf dimensions, while inflorescence length maintained a low CV (18%) (Table 1), suggesting that the latter trait could be subject to stricter genetic regulation in certain populations, in the study of amaranth germplasm in Benin under field conditions with average annual temperatures of 26 °C.

Since this study was conducted under controlled and uniform environmental conditions, the observed phenotypic divergences can be predominantly attributed to the genotypic constitution of the evaluated materials. This interpretation coincides with the conclusions of Nyasulu et al. [10] and Nzungize et al. [11], who suggest that the differentiation in these characters responds to the intrinsic genetic identity of the materials and not to external factors.

In general terms, the high phenotypic diversity identified confirms the potential of amaranth as a valuable resource for targeted selection. According to Yeshitila et al. [6] Singh et al. [18] and Kumari and Khan [19] this wide variation in the gene pool is not only fundamental for the success of genetic improvement programs but is also a critical pillar for the design of long-term plant genetic resource conservation strategies.

The Pearson correlation analysis in this study showed positive and highly significant associations between the vegetative variables, highlighting the codependency between LW and LL (r = 0.99), as well as of the PH with the LL (r = 0.94) and the LW (r = 0.94) (Figure 1). These findings suggest that the increase in the height of the genotypes is directly linked to greater leaf expansion, which coincides with what was reported by Sharma et al. [20] and Yadav et al. [21] carried out in open fields in India, who identified PH and leaf dimensions as critical determinants of leaf biomass in amaranth. In contrast, non-significant negative correlations were observed between vegetative traits and WS, while IL and NL showed phenotypic independence or weak associations (Figure 1). This behavior differs from various previous characterizations where these traits usually show a greater association with performance. For example, Yeshitila et al. [6] reported a negative association between WS and PH, arguing that short genotypes can optimize seed yield through an increase in IL; Simultaneously, Tiwari [22] and Kandel et al. [23] maintain that panicle length and PH are positive predictors of yield. It is possible that greenhouse conditions, by limiting full reproductive development due to suboptimal temperature and lower photosynthetically active radiation, restrict the expression of these correlations between vegetative traits and yield.

Likewise, Chaukse and Vyas [24] emphasize that productivity is linked to the thickness of the stem, the panicle, and the weight of the seeds. In the horticultural field, Ayenan et al. [25], Abrar et al. [26] and Tyrus [27] confirm that leaf area and NL are essential components for green leaf productivity, highlighting the use of amaranth as a vegetable. Finally, the absence of statistical significance in certain associations in this study suggests independence that could be exploited in breeding programs to select specific architectures without compromising seed weight.

The principal component analysis (PCA) identified that the greatest proportion of the phenotypic variation was concentrated in the first component (CP1), defined primarily by PH, LL and LW, while the second component (CP2) grouped the NL and IL (Figure 2). These results present high agreement with those reported by Adedibu et al. [28], who determined that CP1 (30.23% of the variance) integrated leaf dimensions and plant height, while CP2 (11.87%) was associated with the number of leaves and mineral composition. Similarly, Bashyal et al. [29] documented a cumulative variance of 88.3%, where CP1 was positively explained by phenology, height and canopy dimensions, while CP2 grouped NL, Il and the WS. Although there is a convergence with Kpocheme et al. [17] regarding the dominance of the PH, LL and LW in the first variation vector, the present study reveals a structural divergence, since in the aforementioned research the NL and the IL were associated with CP1, while in this work these traits were moved towards CP2 (Figure 2). This discrepancy suggests a differentiation in the partitioning of the phenotypic variance of the materials evaluated, possibly derived from the intrinsic genetic distinction of the genotypes analyzed.

The cluster analysis based on quantitative descriptors allowed discrimination of the evaluated genotypes, identifying that PH, leaf dimensions and number of leaves (NL) were the characters with the greatest influence on the conformation and topology of the clusters (Figure 3). These findings converge with those reported by Bashyal et al. [29], who based the differentiation of four groups on the PH, leaf and petiole dimensions, and by Unlike the findings of Sefasi et al. [16] the suboptimal temperature (16 °C) under which the study was conducted homogenized the yield, prioritizing vegetative traits in the statistical differentiation. Nevertheless, the identification of five clusters confirms significant genetic diversity that can be used for selecting divergent parents to maximize hybrid vigor in plant breeding programs.

The frequency analysis of the qualitative traits revealed a marked polymorphism and phenotypic segregation in the studied population (Figure 4). In the architecture of the young stem, reddish-green pigmentation predominated (73%), while the young leaves exhibited a complex variability, highlighting the dark green coloration with basal pigmentation (45%). During vegetative maturity, ovate leaf morphology (55%), rough veins (82%) and pink petioles (60%) prevailed (Table 2). In the reproductive area, the inflorescences mostly showed an axillary and apical position (82%) with intermediate density (54%) and yellow color (44%), with white being the most common seed color (45%) (Figure 4). These results contrast significantly with the findings of Siamey et al. [30], who evaluated amaranth genotypes under field conditions in Ghana with temperatures above 25 °C and reported, who reported a dominance of green stems (76.2%), foliar homogeneity in green tones (>90%), lanceolate shapes (52.4%) and green petioles (85.7%). Likewise, these authors recorded a higher frequency of green flowers (90.5%) and black seeds (52.4%), differing from the trend observed in this work. This divergence and the wide range of phenotypic diversity identified underline the potential of these materials as elite germplasm. As suggested by Siamey et al. [30], the characterization of these distinctive morphological traits is essential to guide genetic improvement programs towards the specific demands of producers and consumers, optimizing the establishment of characters of agronomic and commercial interest.

The multiple correspondence analysis (MCA) determined that the qualitative variables with the greatest resolving power to explain the phenotypic variance were those associated with the coloration of vegetative and reproductive organs (Figure 5). Specifically, the pigmentation of the foliar, stem, veins, cauline striae, petiole and inflorescence constituted the descriptors with the greatest contribution to intergenotypic differentiation. In contrast, the characters related to the erect growth habit, the presence of striae and pubescence on the stem, as well as the habit of the inflorescence (erect to weakly recurved), were located close to the origin of the factorial plane (Figure 4 and Figure 5). This denotes a low discriminating capacity due to its homogeneous nature within the collection, which limits its usefulness for group segregation. These findings are consistent with what was stated by Yeshitila et al. [12], who identified the pigmentation of stems, leaves and petioles as the determining descriptors in the classification of amaranth genotypes, also observing a predominance of upright habit and inflorescences.

Qualitative cluster analysis classified the 11 genotypes into six distinct groups, whose structure was determined by chromatic polymorphism in vegetative organs and inflorescence traits (Figure 6). In this group, Group I (Genotype 6) stood out, due to its high accumulation of betalain pigments, and Group V (Genotype 9), due to its complex leaf pigmentation pattern with purple mottling and ovoid spots. Although these results partially coincide with what was stated by Yeshitila et al. [12], who grouped 120 genotypes into three clusters based on pigmentation, inflorescence density and presence of thorns, the finding of six groups in a smaller sample evidences a high density of phenotypic variation and genetic heterogeneity. This notable diversity, which proportionally exceeds previous reports, gives high strategic value to the germplasm studied for the selection of parents in genetic improvement programs.

The integral analysis of qualitative and quantitative characters confirms that the variability in amaranth genotypes depends on vegetative pigmentation and morphometric traits (Figure 7 and Figure 8). The presence of betalains and anthocyanins, in addition to being a distinctive marker, offers adaptive advantages; according to Yeshitila et al. [12], these pigments protect against UV-B radiation, function as antioxidants and mediate resistance to biotic and abiotic stress, facilitating selection for extreme climates.

On the other hand, vegetative vigor and photosynthetic capacity are reflected in the height of the plant and leaf dimensions. In this regard, Yeshitila et al. [6] point out that a greater leaf area optimizes the production of photoassimilates, which favors greater seed caliber and yield, especially in late flowering genotypes.

Although variables such as plant height, panicle length and seed weight are standard predictors of productive potential [22,23,28,31], the yield projection based on a commercial density of 260,417 plants/ha [15] should be considered a Comparative ranking under controlled conditions. These estimates may vary in open fields due to the interaction between agronomic management and environmental factors.

Within the evaluation, genotype 10 stood out as the most promising with an estimated yield of 642.94 kg/ha (Figure 9), the highest weight of a thousand seeds (0.91 g) and an inflorescence length of 21.72 cm. This performance is consistent with what was stated by Bashyal et al. [29], who associate the simultaneous improvement in panicle length and seed weight with higher yield. Likewise, Sefasi et al. [16] reinforce that genotypes that integrate superior values in performance and morphological characters are the ideal candidates for genetic improvement programs.

On the other hand, genotypes 2 and 8 stood out for their versatility and efficiency (Figure 9). Genotype 2 (532.99 kg/ha) presented superior vegetative vigor (73.89 leaves and 65.06 cm in height), an ideal profile for dual use (grain and forage), while genotype 8 combined good performance with a size of 62.36 cm and a weight of 1000 seeds of 0.82 g, which according to Yeshitila et al. [6] is commercially desirable to mitigate the risk of lodging.

At the lower end, genotypes 1 and 7 presented the lowest yields (242.48 and 239.29 kg/ha), consistent with their lowest values of plant height (38.19 and 36.09 cm) and leaf dimensions (Figure 9). However, genotype 1 exhibited the second highest 1000 seed weight (0.89 g), indicating that its low overall yield is due to lower seed production per plant and not to poor or reduced grain size, giving it strategic value as an allele donor for grain size in targeted hybridization programs.

Finally, the yields estimated in the present study exhibit a magnitude significantly lower than the standards documented in open field conditions. In commercial production systems with optimized agronomic management, grain productivity in amaranth usually ranges between 1500 and 7200 kg/ha, depending on the cultivar and the agroclimatic variables of the environment [32]. This discrepancy was expected, given that the greenhouse environment, although it guarantees the experimental homogeneity necessary to determine the relative genetic potential, does not accurately replicate determining factors such as the incidence of photosynthetically active solar radiation, the dynamics of competition in real planting densities or the management practices typical of large-scale agriculture.

Consequently, the values obtained constitute a comparative ranking of intergenotypic performance under standardized conditions but should not be interpreted as absolute parameters of commercial performance. Under this premise, the validation of genotypes 10, 2 and 8 is recommended through multilocal field trials that contemplate the direct determination of reproductive biomass. These subsequent studies are imperative to corroborate the productive superiority and phenotypic stability of these materials in the face of biotic and abiotic factors of real cultivation systems.

## 4. Materials and Methods

### 4.1. Study Area

The morpho-agronomic characterization was carried out under protected environment conditions (greenhouse) at the main campus of the Pedagogical and Technological University of Colombia (UPTC), located in the municipality of Tunja, Boyacá. The study area is geographically located at 5° 33′ North latitude and 73° 24′ West longitude, at an altitude of 2691 m above sea level. During the experimental period, an average temperature of 16.2 °C and a relative humidity of 71.8% were recorded [33].

### 4.2. Plant Material

Eleven amaranth varieties (Table 3) from the Boyacá Governorate’s seed collection were evaluated using qualitative and quantitative descriptors. The 11 cultivars were specifically selected for their presence and availability in the department of Boyacá, Colombia. The selection focused on their geographic relevance, prioritizing materials already available in the region to address the technical gap in local amaranth production. Regarding diversity of uses, the selection sought to encompass various categories, including grain-producing species and those with potential for other agricultural applications. These varieties were sown in peat germination trays, and the seedlings were then transplanted into polyethylene bags containing a substrate composed of rice hulls and black soil in a 20:80 ratio in the greenhouse of the Pedagogical and Technological University of Colombia.

### 4.3. Experimental Design

The study was established under a Randomized Complete Block Design (RCBD). Eleven amaranth genotypes were evaluated as treatments, with three blocks (replicates) used to control for environmental gradients and internal variability within the greenhouse. Each experimental unit within a block consisted of three individual plants (sub-samples) per genotype. Consequently, the total experimental population comprised 99 observation units (11 genotypes × 3 blocks × 3 plants per unit).

The blocks were arranged orthogonally to the main source of environmental variation (such as light or ventilation gradients) to ensure that intra-block variability was minimized. Agronomic management, including manual weeding, localized fertilization, and irrigation, was applied uniformly according to the specific needs of the crop. Harvesting and threshing were conducted manually for each individual plant upon reaching physiological maturity.

### 4.4. Morphoagronomic Characterization

The morpho-agronomic characterization of amaranth cultivars was performed using 19 qualitative descriptors and 6 quantitative variables (Table 2), standardized by the International Union for the Protection of New Varieties of Plants [34], the graphic manual for varietal description in amaranth [35], and characterization protocols validated in previous studies [6,10,11,29]. The two descriptors were recorded at two phenological stages, 3 and 5 months after sowing (Table 4).

### 4.5. Data Analysis

For the analysis of the data obtained in the morpho-agronomic characterization, descriptive statistics, and Tukey’s test for comparison with *p* < 0.05 were performed to determine the significant differences between the cultivars, using the statistical software R version 4.5.2. For the qualitative variables, frequency analysis was carried out in the statistical software InfoStat 2020. Pearson correlation analyses for the quantitative variables were performed with the package “corrplot”: Visualization of a Correlation Matrix (Version 0.84) [36].

For multivariate analysis, hierarchical principal component analysis (HCPC) was performed using the algorithms included in the Factoextra package of the R program, which were represented in a two-dimensional plane using the FactoMineR package. Euclidean distances and Ward’s minimum distances were considered in the cluster analyses using FactoMineR. For mixed analysis, considering both qualitative and quantitative variables, mixed principal component analysis (PCAmix) was employed, implemented using the PCA mixdata package of R [37].

For the selection of superior genotypes, the total weight of seeds obtained from nine plants per genotype was recorded. The data were extrapolated to estimate the potential yield (kg/ha), considering a population density of 260,417 plants/ha, according to the parameters established for commercial crops in Mexico by Romero et al. [15].

## 5. Conclusions

The morphoagronomic characterization allowed us to determine a wide genetic variability in the evaluated germplasm, achieving the segregation of the genotypes into differentiated groups. This diversity, manifested in both quantitative traits (height and leaf dimensions) and qualitative traits (chromatic polymorphism due to betalains), constitutes a solid basis for identifying genetic relationships and guiding selection processes within the breeding program.

The multivariate analysis revealed that performance does not depend on a single variable but on an integration of factors. It is concluded that, in the population studied, the characteristics of the leaf and the height of the plant are closely linked to vegetative vigor, while pigmentation acts as a determining descriptor for the differentiation between genotypes and the potential for adaptability to stress conditions.

Genotypes 10, 2 and 8 were consolidated as materials with the greatest agronomic potential. Genotype 10 stood out for its superiority in estimated yield and weight of 1000 seeds, while genotypes 2 and 8 exhibited an optimal balance between vegetative vigor and reproductive efficiency, positioning themselves as ideal candidates for incorporation into genetic improvement schemes for the species.

## Figures and Tables

**Table 1:** Descriptive statistics of the quantitative variables evaluated for the characterization of the 11 amaranth cultivars in Tunja, Boyacá.

Genotype n = 11	PH	NL	LL	LW	IL	WS
Gen1	38.19	59.11	5.54	2.93	25.75	0.89
Gen2	65.06	73.89	7.17	3.52	29.59	0.79
Gen3	76.44	43.56	11.80	6.67	34.31	0.57
Gen4	65.31	50.78	10.36	6.03	21.97	0.81
Gen5	56.30	51.22	6.40	4.23	34.30	0.85
Gen6	55.51	61.11	9.90	4.98	22.22	0.48
Gen7	36.09	19.04	2.87	1.48	29.56	0.76
Gen8	62.36	48.22	7.59	4.00	20.62	0.82
Gen9	67.39	56.33	8.85	3.97	25.48	0.38
Gen10	64.00	48.56	8.69	4.31	21.72	0.91
Gen11	71.33	41.22	7.60	4.38	32.29	0.57
Average	59.82	50.28	7.89	4.23	27.07	0.71
CV %	21.21	27.42	31.21	33.15	19.14	25.15
Σ	12.69	13.79	2.46	1.40	5.18	0.18

Genotype Gen; PH: Plant height (cm); NL: Number of leaves; LL: Leaf length (cm); LW: Leaf width (cm); IL: Inflorescence length (cm); WS: Weight of 1000 seeds (g). σ: Standard deviation; CV: Coefficient of variation.

**Table 2:** Frequency analysis for the qualitative descriptors used for the characterization of the 11 amaranth cultivars in Tunja, Boyacá.

Descriptor	Dominant Category	F. Absolute	F. Relative (%)
Growth habit (GH)	Erect	99	100
Stem pubescence (SP)	Present	90	91
Pigmentation of the young stem (PYS)	Reddish green	72	73
Pigmentation of the adult stem (PAS)	Red-pink	41	41
Streaks on the stem (ST)	Present	99	100
Color of the striations on the stem (CST)	Red	47	47
Pigmentation in young leaves (PYL)	Dark green blade, with pigmentation at the base of the upper third of the leaves.	45	45
Pigmentation in adult leaves (PAL)	Green	25	25
Leaf shape (LS)	Ovada	54	55
Margin of Leaf (ML)	Entire	74	75
Color of the veins of the leaves (LVC)	Green	83	84
Prominence of veins (PV)	Rough	81	82
Pigmentation of the petiole (PPE)	Pink	59	60
Position of the inflorescence (PI)	Axillary and apical inflorescence	81	82
Type of inflorescence (TI)	Glomerular	78	79
Inflorescence density (ID)	Media	53	54
Inflorescence shape (IS)	Erect or weakly recurved	90	91
Color of the inflorescence (CI)	Yellow	44	44
Seed color (SC)	White	45	45

**Table 3:** Amaranth genetic material evaluated morpho-agronomically under controlled conditions in Tunja, Boyacá.

Code	Country of Origin	Collection Site	Harvest Date	Georeferencing	Common Name	Species
BGQ0030 (Gen1)	Colombia	Agricultural Diagnostic Center	2013	5°33′13.8″ N 73°21′37.1″ W	Kiwicha white seed	*A. cruentus*
BGQ0051 (Gen2)	Ecuador	Ecuador-INIAP		0°22′07.3″ S 78°33′18.5″ W	Kiwicha INIA Alegría	*A. cruentus*
BGQ0039 (Gen 3)	Colombia	Agricultural Diagnostic Center	2014	5°33′13.8″ N 73°21′37.1″ W	Kiwicha black	*A. hypochondriacus*
NA (Gen4)			2018		Kiwicha INIAP alegría—Harvested January 2018	*A. hypochondriacus*
BGQ0076 (Gen5)	Colombia	Nariño	2013		Kiwicha	*A. cruentus*
NA (Gen6)	Colombia	Subachoque-Cundinamarca	2020	4°55′41″ N 74°10′25″ O	Black amaranth	*A. caudatus*
NA (Gen 7)	Colombia	Subachoque-Cundinamarca	2020	4°55′41″ N 74°10′25″ O	White amaranth	*A. cruentus*
BGQ0021 (Gen 8)	Colombia	Agricultural Diagnostic Center	2017	5°33′13.8” N 73°21′37.1″ W	White kiwicha	*A. hypochondriacus*
NA (Gen9)	Colombia	Subachoque-Cundinamarca	2024	4°55′41″ N 74°10′25″ O	Black amaranth	*A. hypochondriacus/A. hybridus*
BGQ0023 (Gen10)	Colombia	La Colorada Tunja	2017	5°34′44.7″ N 73°20′36.0″ W	Kiwicha	*A. hypochondriacus*
BGQ0010 (Gen11)	Colombia	Agricultural Diagnostic Center	2013	5°33′13.8″ N 73°21′37.1″ W	Kiwicha	*A. hypochondriacus*

**Table 4:** List of qualitative and quantitative morphological descriptors used in the characterization of amaranth cultivars (*Amaranthus* spp.) under controlled conditions in Tunja, Boyacá.

Qualitative	Abbreviation	Descriptor and Code
Growth habit	GH	1 = Upright, 2 = Prostrate
Stem pubescence	SP	1 = Absence, 2 = Presence
Pigmentation of the young stem	PYS	1 = Green, 2 = Yellow, 3 = Pink, 4 = Red, 5 = Purple, 6 = Reddish-green, 7 = Reddish-pink
Pigmentation of the adult stem	PAS	
Streaks on the stem	ST	1 = Absence, 2 = Presence
Color of the striations on the stem	CST	1 = Red, 2 = Purple, 3 = Green, 4 = Yellow, 5 = None
Pigmentation in the young leaves	PYL	1 = Green, 3 = Entire leaves purple, 4 = Margin and veins pigmented, 5 = Ovoid central spot with pigmented margin and veins, 6 = Entire leaf green with pigmented base, 7 = Purple mottling with pigmented margin and veins, 8 = Dark green, 9 = Entire leaf green with pigmented margin, 10 = Entire leaf reddish-pink, 11 = Reddish-green blade, 12 = Dark green leaf, with pigmentation at the base of the upper third of the leaves
Pigmentation in the adult leaves	PAL	
Leaf shape	LS	1 = Lanceolate, 2 = Elliptical, 3 = Cuneiform, 4 = Obovate, 5 = Ovate, 6 = Oval, 7 = Rhomboid, X = Mixed
Margen of leaf	ML	1 = Entire, 2 = Crenate, 3 = Wavy, 4 = Sinuous, X = Mixture
Leaf vein color	LVC	1 = Green, 2 = Pink, 3 = Red, 4 = Purple
Prominence of veins	PV	1 = Smooth, 2 = Rough
Pigmentation of the petiole	PPE	1 = Green, 2 = Pink, 3 = Amaranth, 4 = Red, 5 = White, 6 = Yellow
Position of the inflorescence	PI	1 = Terminal inflorescence, 2 = Axillary and apical inflorescence
Type of inflorescence	TI	1 = Amarantiform, 2 = Glomerulated
Inflorescence density	ID	3 = Loose, 5 = Medium, 7 = Dense
Inflorescence shape	IS	1 = Erect or weakly recurved, 2 = Intermediate, 3 = Strongly recurved
Color of the inflorescence	CI	1 = Yellow, 2 = Green, 3 = Pink, 4 = Red, 5 = Purple, 6 = Brown, 7 = Red-pink
Seed color	SC	1 = White, 2 = Yellow, 5 = Black
**Quantitative**	**Abbreviation**	
Plant height (cm)	PH	
Number of leaves	NL	
Leaf length (cm)	LL	
Leaf width (cm)	LW	
Inflorescence length (cm)	IL	
Weight of 1000 seeds (g)	WS	

## Data Availability

The original contributions presented in this study are included in the article/Supplementary Materials such as the original databases, the tables of the multivariate analyses, as well as additional information on the groupings formed in the different analyses carried out in the study. Further inquiries can be directed to the corresponding author.

## Supplementary Materials

The following supporting information can be downloaded at https://www.mdpi.com/article/10.3390/plants15111648/s1: Table S1: Summary of hierarchical clustering analysis for qualitative descriptors.; Table S2: Summary of hierarchical clustering based on the integration of quantitative and qualitative descriptors. Table S3. Mixed principal component analysis for the 11 amaranth genotypes evaluated. Table S4. Summary of the main morphological characteristics that define the groupings of the 11 amaranth genotypes evaluated. Table S5. Summary of the main qualitative and quantitative variables that define the groupings in the mixed UPGMA analysis.

## Abbreviations

The following abbreviations are used in this manuscript:

GH	Growth habit
SP	Stem pubescence
PYS	Pigmentation of the young stem
PAS	Pigmentation of the adult stem
ST	Streaks on the stem
CST	Color of the striations on the stem
PYL	Pigmentation in the young leaves
PAL	Pigmentation in the adult leaves
LS	Leaf shape
ML	Margin of leaf
LVC	Leaf vein color
PV	Prominence of veins
PPE	Pigmentation of the petiole
PI	Position of the inflorescence
TI	Type of inflorescence
ID	Inflorescence density
IS	Inflorescence shape
CI	Color of the inflorescence
SC	Seed color
PH	Plant height (cm)
NL	Number of leaves
LL	Leaf length (cm)
LW	Leaf width (cm)
IL	Inflorescence length (cm)
WS	Weight of 1000 seeds (g)
