# Supplementary material for: Identification of Promising Amaranth (Amaranthus spp.) Genotypes Through Multivariate Analysis of Quantitative and Qualitative Descriptors in Boyacá
Source: Plants (Basel). 2026 May 27;15(11):1648. doi: 10.3390/plants15111648 (PMC13259516; doi:10.3390/plants15111648)
Supplement: Supplementary file 1 [file plants-15-01648-s001.zip › plants-4306546-supplementary.pdf]

Table S1. Principal component analysis for the quantitative variables evaluated in the 11 amaranth genotypes.

	PC1	PC2	PC3	PC4	PC5	PC6
**PH**	0,51	-0,15	-0,18	0,05	0,82	0,09
**NL**	0,24	0,53	-0,18	-0,79	-0,06	0,06
**LL**	0,56	0,09	0,02	0,18	-0,26	-0,76
**LW**	0,53	-0,01	-0,22	0,29	-0,46	0,61
**IL**	-0,02	-0,73	-0,51	-0,37	-0,19	-0,15
**WS**	-0,29	0,39	-0,79	-0,79	0,08	-0,13

PH: Plant height (cm); *NL*: Number of leaves; *LL*: Leaf length (cm); *LW*: Leaf width (cm); *IL*: Inflorescence length (cm); *WS*: Weight of 1000 seeds (g).

Table S2. Multiple correspondence analysis for the qualitative variables evaluated in the 11 amaranth genotypes.

	PC1	PC2	PC3
**GH_1**	0,00	0,00	0,00
**SP_1**	-0,37	1,65	0,10
**PYS_2**	0,04	-0,17	0,01
**PYS_1**	-0,30	1,77	0,11
**PYS_5**	3,14	0,08	-0,32
**PYS_6**	-0,32	-0,45	0,01
**PAS_2**	-0,30	1,77	0,11
**PAS_3**	-0,34	0,57	0,10
**PAS_5**	-0,27	-1,15	-0,10
**PAS_6**	-0,25	-0,45	1,09

GH: Growth habit; *SP: Stem pubescence*; *PYS:* Pigmentation of the young stem; *PAS:* Pigmentation of the adult stem.

Table S3. Mixed principal component analysis for the 11 amaranth genotypes evaluated.

	PC1	PC2
**PH**	0,09	0,84
**NL**	0,11	0,00
**LL**	0,38	0,42
**LW**	0,24	0,43
**IL**	0,09	0,01
**WS**	0,45	0,05
**SP**	0,14	0,24
**PYS**	0,91	0,83
**PAS**	0,34	0,65
**CST**	0,84	0,60
**PYL**	0,96	0,79
**PAL**	0,96	0,82
**LS**	0,00	0,77
**ML**	0,03	0,03

**LVC**	0,85	0,22
**PV**	0,10	0,15
**PPE**	0,88	0,59
**PI**	0,61	0,02
**TL**	0,21	0,47
**ID**	0,04	0,11
**IS**	0,79	0,18
**CI**	0,91	0,47
**SC**	0,48	0,17

PH: Plant height (cm); *NL*: Number of leaves; *LL*: Leaf length (cm); *LW*: Leaf width (cm); *IL*: Inflorescence length (cm); *WS*: Weight of 1000 seeds (g)*; SP*: *Stem pubescence; PYS:* Pigmentation of the young stem; *PAS*: Pigmentation of the adult stem; *CST*: Color of striations on the stem; *PYL*: Pigmentation of young leaves; *PAL*: Pigmentation of adult leaves; *LS*: Leaf shape; *ML*: Leaf margin; *LVC*: Color of the veins in the leaves; *PV*: Prominence of veins; *PPE*: Pigmentation of the petiole; *PI*: Position of the inflorescence; *TL*: Type of inflorescence; *ID*: Inflorescence density; *IS*: Inflorescence shape; *CI*: Color of the inflorescence; *SC*: Seed color.

Table S4. Summary of the main morphological characteristics that define the groupings of the 11 amaranth genotypes evaluated.

Cluster	Genotypes	Descriptor	Seed color
**I**	1, 7	Yellow stem / Yellow inflorescence	White
**II**	2, 5	Reddish-green stem / Pink inflorescence	White/Yellow
**III**	6	**Maximum pigmentation (Purple/Red-pink)**	Black
**IV**	4, 8, 10	Ovate leaf / Red streak / Dense inflammation	White/Yellow
**V**	9	**High morpho-chromatic variability**	Black
**VI**	3, 11	Purple inflorescence / Red streak	Black

Table S5. Summary of the main qualitative and quantitative variables that define the groupings in the mixed UPGMA analysis.

Cluster	Genotypes	Key Qualitative Descriptor	Relevant Quantitative Attribute
**I**	6	Purple Pigmentation / Terminal Inflammation	Total morphological divergence
**II**	1, 7	Yellow Stem / Yellow Inflammation / White Seed	Minimum values of vegetative vigor
**III**	2, 5	Infl. Pink	Intermediate values of vegetative vigor
**IV**	4, 8, 10	Red Stretch Marks / Red-Pink Stem / Infl.Yellow	High vegetative vigor
**V**	9	Pink Veins / Type 4 Pigmentation	Minimum weight of one thousand seeds (0.38 g)
**VI**	3, 11	Infl. Purple / Red Streak/ Black Seed	Max. values for height and inflorescence length, same weight of one thousand seeds (0.57g)
